# Ethics rounds in the ambulance service: a qualitative evaluation

**DOI:** 10.1186/s12910-024-01002-6

**Published:** 2024-01-18

**Authors:** Catharina Frank, Andreas Rantala, Anders Svensson, Anders Sterner, Jessica Green, Anders Bremer, Bodil Holmberg

**Affiliations:** 1 Faculty of Health and Life Sciences, Linnaeus University, Växjö, Sweden; 2 Centre of Interprofessional Collaboration within Emergency care (CICE), Linnaeus University, Växjö, Sweden; 3 Department of Health Sciences, Faculty of Medicine, Lund University, Lund, Sweden; 4 Department of Ambulance Service, Region Skåne, Helsingborg, Sweden; 5 Faculty of Caring Sciences, Work Life and Social Welfare, University of Borås, Borås, Sweden; 6 Department of Ambulance Services, Region Kalmar County, Kalmar, Sweden; 7 Department of Health Sciences, Red Cross University College, Stockholm, Sweden; 8 Department of Ambulance Service, Region Kronoberg, Sweden; 9 Department of Health and Caring Sciences, Faculty of Health and Life Sciences, Linnaeus University, Växjö, SE-352 52 Sweden

**Keywords:** Ambulance clinicians, Ethics rounds, Intervention, Qualitative, Evaluation, Ethical competence, Decision-making, Patient autonomy

## Abstract

**Background:**

It is a common ethical challenge for ambulance clinicians to care for patients with impaired decision-making capacities while assessing and determining the degree of decision-making ability and considering ethical values. Ambulance clinicians’ ethical competence seems to be increasingly important in coping with such varied ethical dilemmas. Ethics rounds is a model designed to promote the development of ethical competence among clinicians. While standard in other contexts, to the best of our knowledge, it has not been applied within the ambulance service context. Thus, the aim of this study was to describe ambulance clinicians’ experiences of participating in ethics rounds.

**Methods:**

This was a qualitative descriptive study, evaluating an intervention. Data were collected through sixteen interviews with ambulance clinicians who had participated in an intervention involving ethics rounds. The analysis was performed by use of content analysis.

**Results:**

Two themes describe the participants’ experiences: (1) Reflecting freely within a given framework, and (2) Being surprised by new insights. The following categories form the basis of the themes; 1a) Gentle guidance by the facilitator, 1b) A comprehensible structure, 2a) New awareness in the face of ethical problems, and 2b) Shared learning through dialogue.

**Conclusion:**

Incorporating structured ethics rounds seems to create a continuous development in ethical competence that may improve the quality of care in the ambulance service. Structured guidance and facilitated group reflections offer ambulance clinicians opportunities for both personal and professional development. An important prerequisite for the development of ethical competence is a well-educated facilitator. Consequently, this type of ethics rounds may be considered a useful pedagogical model for the development of ethical competence in the ambulance service.

**Supplementary Information:**

The online version contains supplementary material available at 10.1186/s12910-024-01002-6.

## Introduction

In Sweden, the Advanced Life Support ambulances are staffed by a two-person team comprising at least one registered nurse (RN). The other team member is an emergency medical technician, RN, or a specialist trained RN. Working in the ambulance service involves dealing with numerous ethical challenges, i.e., ethically problematic situations, characterized by multidimensional suffering, which requires the ability to provide medical care while establishing a trusting caring relationship [1]. These ethical challenges manifest in various areas, including the facilitation of patient autonomy for patients with impaired decision-making ability, the accommodation of different cultural traditions and values, and working under the risk of physical violence or verbal abuse [2], indicating that ambulance clinicians routinely encounter ethical dilemmas in complex situations. Simultaneously, the emergency medical services in many western countries are facing an increasing number of frail older patients [3–5]. In order to cope with an increasing proportion and variety of ethical dilemmas, the ambulance clinicians’ ethical competence appears to be increasingly important [6], however, sometimes this ethical competence is lacking [7]. Consequently, it is important to support the clinicians’ abilities and development in ethical competence, which may be achieved, e.g., by introducing ethics rounds (8–9).

## Background

Ambulance clinicians face ethical problems, make decisions, and solve problems that are different from clinical work. Also, there is a lack of interprofessional support on site and the clinicians must make independent and responsible decisions within short time-frames. In this context, ethically problematic care situations involve areas such as withholding resuscitation and end-of-life care, triage, child abuse, refusal of treatment, delay or denial of transport for non-emergent conditions, patients’ decision-making capacity, and patients’ self-determination [1].

Patient assessments within the ambulance service are performed using structured support models to determine the medical symptomatology, which is subsequently addressed with appropriate treatment [10]. However, the assessment of what is happening with the patient behind this symptomatology is typically less structured and relies on healthcare expertise with a focus on the patients’ needs within the broader perspective, rather than just addressing the medical necessities [10]. This has highlighted the need to enable measures and attitudes to foster patient security, trust, and participation [11], and to counteract suffering from the care itself, or from lack of care [12]. In addition, assessing patients is challenging, particularly when they are suffering from acute illnesses that result in non-specific or unusual symptoms [13]. Moreover, as people age, their ability or willingness to make decisions during acute health problems is often impaired [14] and sometimes completely absent [15]. When patients, regardless of their age, are unable to express themselves, this can have direct consequences for their autonomy, integrity, and dignity [16]. In such cases, ambulance clinicians must rely on some form of proxy decision-making, often with the support of information provided by significant others. However, this is sometimes based solely on the clinicians’ assessment of what is best for the patient, in relation to symptoms, observed vital signs, and any other available information [17]. In this context, the importance of ambulance clinicians having knowledge and understanding of the ethical aspects of proxy decision-making has been emphasised, as this can affect the patient’s well-being and autonomy [14].

As populations age, older patients with impaired decision-making abilities pose a common challenge for ambulance clinicians when assessing and determining the degree of decision-making ability, choosing potential areas for shared decision-making, balancing ethical values, and relating to their own ethical values [14]. Within an ambulance service, this requires ambulance clinicians to provide empathetic care to patients and their significant others, based on ethical values and norms that aim to promote well-being, life, and justice, and demonstrate respect for self-determination and integrity [18].

One method designed to promote the development of ethical competence among clinicians is through the implementation of ethics rounds [19], and this approach has been adopted within various healthcare settings [20–22], albeit, to our knowledge, not within the ambulance service context. The primary purpose of ethics rounds is to support healthcare clinicians in coping with ethically challenging situations by examining specific situations that are raised by one or more clinicians [9, 23]. Ethics rounds are led by a designated facilitator, preferably an ethicist with expertise in facilitating such discussions [24] but can also be led by clinically active individuals with or without specialized knowledge in ethics [21]. The facilitator’s role is to promote an open dialogue and encourage group reflection [24]. Moral case deliberations, including ethics rounds, usually have 8–12 participants, including the case presenter and the facilitator [25].

In summary, patients with impaired decision-making abilities present a challenge for ambulance clinicians when it comes to ensuring important ethical values in care and nursing. Consequently, ambulance clinicians need to train and develop their ability to critically reflect on and evaluate the ethical values that are at stake in every unique situation they encounter in clinical practice. Thus, the aim of this study was to describe ambulance clinicians’ experiences of participating in ethics rounds.

## Methods

### Design

This was a qualitative interview study with the objective of evaluating an intervention comprising ethics rounds.

### Intervention

The intervention was part of the research project “Ethically good care for older persons with acute health problems” (ECA), which had the overall aim of preventing the unfair treatment of older patients who have been afflicted by acute disease, injury, or illness. In the project, an intervention was carried out to strengthen the ethical competence of the ambulance staff. The objectives of ethical reflection near practice can comprise, for example, raising the level of ethical competence, which includes supporting values such as fairness, self-determination, and beneficence. The consensus in ethics reflection groups is that no one individual is an expert who possesses all the answers. Ethical reflection promotes the skills to identify, reflect upon, and deal with demanding ethical problems in everyday situations [26].

Before the ethic rounds, all participants were shown a main video and two short subsequent videos that revolved around an older couple facing a relatively common problem concerning a general decline. In the video scenario, the ambulance clinicians wanted the male patient to accompany the ambulance clinicians to the hospital, while he himself did not want to go. The main video depicted a scenario that included all parties involved, while the subsequent shorter videos focused on individual conversations that presented varying degrees of ethical competence shown by the ambulance clinicians in their interactions with the older couple.

The ethics rounds, were led by a facilitator who was a medical ethicist, educated in the 6-step model [26] and experienced in leading ethical reflections. The 6-step model is interactive, with participants being asked to provide input to the six steps; (1) What is the ethical challenge? (2) What are the facts of the case? (3) Who are the parties involved, and what are their views and interests? (4) Which values, principles and legislation come into play? (5) What are the possible courses of action? and (6) Holistic discussion/evaluation of courses of action. Although the steps are usually discussed in numerical order, the model allows for going backwards and forwards across the steps. The facilitator summarized the group discussion by completing the worksheet on a large screen visible to the participants in real-time during the ethics rounds. In line with recommendations in the model, the group was told that the facilitator would not provide the ethical answer to their situation. Rather, the role of the facilitator was to help the group articulate their thoughts and assist them in the process. There was, however, an agreement at the outset of the ethics round that the facilitator would object if the group should reach any obviously unethical conclusions.

The ethics rounds were conducted at ambulance stations in southern Sweden. In total, 78 ambulance clinicians (94% RNs and 6% emergency medical technicians) participated in a total of 14 ethics rounds, i.e., on average, 6 clinicians participated in each round (range 3–7). The participants gathered for two hours in each ethics round to systematically examine the ethical challenges that they perceived in the video clips. Six groups met digitally and eight met face-to-face. Those who met digitally worked in different regions and were unknown to each other, while those who met face-to-face knew each other as they worked at the same ambulance station.

### Participants and research context

Those who took part in the interviews in this present study were ambulance clinicians who had participated in the intervention, i.e., in one ethics round, either face-to-face or digitally.

The inclusion criteria stipulated that the ambulance clinicians should be actively employed in their respective professions, including ambulance nurses, paramedics, and registered nurses. Participants in the ethics rounds were asked whether they were willing to take part in a subsequent interview study regarding their personal experiences of the ethics rounds at a later stage.

The participants were drawn from two regions in southern Sweden. All 16 participants were registered nurses, whereof 14 had specialist training within various areas (Table 1). The participants had worked in the ambulance service for a period ranging from two to 25 years (mean 10.5 years).

In total, 16 nurses agreed to participate, including nine men and seven women. The age distribution ranged from 31 to 65 years (mean age 43 years).

**Table 1:** Demographics of the ambulance clinicians (*n* = 16)

	RN^a^ (*n* = 2)	PEN^b^ (*n* = 13)	AN^c^ (*n* = 1)
Men, *n*	2	6^d^	1
Women, *n*	0	7^e^	0
Age, years, *median (range)*	48 (31–65)	43 (31–58)	45
Working experience in ambulance service, years, *median (range)*	17.5 (10–25)	9.5 (2–24)	19

^a^ Registered Nurse without specialist education (RN), ^b^ Prehospital Emergency Nurse (PEN), ^c^ Anaesthesia Nurse (AN), ^d^ One PEN was also a specialist Public Health Nurse, ^e^ One PEN was also a specialist Geriatric Nurse

### Data collection

Approximately six months after the ethics round intervention, the data collection was carried out by open-ended individual interviews that were conducted between May and August 2022. The opening question was: How did you experience the ethics round? Subsequently, the interviewers posed questions covering the impact of the ethics round regarding ethical competence, autonomy, paternalism, moral courage, stress, and workplace climate. Further, follow-up questions were then asked, such as: “What do you mean?”, and “In what way?” (see supplementary file, interview guide). Each interview lasted between 22 and 55 min (median 36 min) and was digitally audio-recorded. The data consisted of 16 interviews, which were transcribed verbatim by a professional language and translation agency.

### Data analysis

The interviews were analyzed by J.G. and then further analyzed by B.H. and C.F. The chosen method was qualitative content analysis aiming at the generation of categories and themes [27]. The initial step was to read through the interviews multiple times to get a sense of the whole. Subsequently, a stepwise analysis of the data was initiated by identifying meaning units. The meaning units were then condensed, where the essential message from the units of meaning was preserved. After that, the condensed meanings were coded and furthermore developed into categories. This was achieved by comparing and interpreting between the categories, leading to the identification and creation of latent themes (Table 2).

**Table 2:** Example of the analysis process

Meaning unit	Condensed meaning unit	Code	Category	Theme
And then it was positive that you received guidance. After all, we had (…) who guided us and like … It’s so easy to start off on the wrong track, but he always steered us right into line and that we kept to the point.	Positive with guidance so the conversation is steered correctly, and you keep it to the point so you don’t drift off.	Guidance	A gentle guidance by the facilitator	Reflecting freely within a given framework

### Ethical considerations

Throughout the research process the principles of the Declaration of Helsinki [28] were considered and applied. All participants were informed that their participation was voluntary and that they could withdraw at any time without providing a reason, and each signed a written consent form prior to the data collection. Permission was granted by the Swedish Ethical Review Authority prior to the study (No. 2021–03490).

## Results

The results consist of two themes that describe the participants’ experiences: (1) Reflecting freely within a given framework, and (2) Being surprised by new insights. The following categories form the basis of the themes; 1a) Gentle guidance by the facilitator, 1b) A comprehensible structure, 2a) New awareness in the face of ethical problems, and 2b) Shared learning through dialogue.

### Reflecting freely within a given framework

The structure of the ethics rounds conveyed an opportunity for free reflection within a given framework, which was described as a positive and revolutionary experience. A prudent process of guidance created an open-minded and permissive atmosphere where the courage to express thoughts within the group was encouraged. The well-known video scenarios that were discussed during the ethics rounds constituted a comprehensible and uncomplicated basis for encouraging joint reflections.

#### Gentle guidance of the facilitator

The participants described the ethics rounds as being carried out with the help of gentle guidance provided by the facilitator. This signified a simultaneously firm and careful guidance, which was about controlling the time so that all participants had the opportunity to speak. The gentle guidance was also described as being a cautious guide of the discussion that raised and deepened various ethical problems from the perspective of each participant. The facilitator was described as having experience of this type of guidance, being used to leading discussions, and possessing a solid knowledge base about ethics. The gentle guidance was carried out in an open-minded way with a permissive approach, which created a good atmosphere and the feeling that there were various ways of reasoning:

*No one was being exposed for thinking wrong or not getting it or being stupid … it was a very open climate.* (Interview No 4)

This atmosphere was of great importance to the participants, who then felt safe and encouraged to freely talk about their own experiences, thoughts, and feelings. It was appreciated that the facilitator firmly sought detailed answers from all participants, including those known to be characteristically quiet in their everyday context. Further, it was described as being important that all participants were given the necessary time to answer questions about their reasoning and actions in ethically difficult situations, which was instructive for everyone listening. The gentle guidance contributed to an open conversation climate, where different opinions could be discussed in a respectful manner, whereas the facilitator remained calm within the presentation of all types of answers and behaviors:

*He (the facilitator) led us in a factual and professional way and helped us put what we expressed into the right box, still reaching a common solution in the end.* (Interview No 15)

The questions posed to the participants during the ethics rounds were perceived as being relevant and friendly, which contributed to a permissive atmosphere that enabled the further development of the ethical problems discussed.

#### A comprehensible structure

The ethics rounds were described as following a comprehensible structure in terms of following their steps and content. The number of participants in the group was described as being sufficient for enabling reasoning, allowing everyone to participate, and it was perceived as being a positive element that there was time set aside for the ethics rounds without being disturbed, such as sudden callouts for ambulance or phone calls. The structure was described as promoting the opportunity to freely discuss and immerse oneself into the ethical problems, despite conflicting views:

*It was a lot about exchanging experiences and like: I probably would have done it this way, and: I understand how you think, but I would probably have done like that /…/ If I have one opinion and another had a different opinion, we still had to resolve the situation*. (Interview No 10)

It was valuable for participants to know each other, as this felt safe and contributed to a conversation similar to those conversations recognized in the everyday work at the ambulance station. However, some described it as an advantage when participants did not know each other, as it was easier to share opinions regarding choice of actions with strangers. The lack of a close working relationship with the other participants then meant an increased sense of freedom to express divergent opinions, especially if these could be assumed to deviate from the opinions of the group. Other benefits were that the participants could vary greatly in terms of experience, age and education, which contributed to the depth of the conversation. Further, participating in digital ethics rounds with colleagues from other, unknown workplaces was described as being particularly rewarding, as these provided otherwise unknown ways of solving ethical problems.

The starting point, with a fictional scenario in a video, mirroring a situation recognizable to all participants, motivated the ambulance clinicians to actively listen and participate in the discussion. Building the discussion on a fictional scenario was described as an advantage compared to the use of authentic cases shared by one of the participants, as this gave all the participants the same conditions and starting point in the subsequent discussion. In addition, the risk of criticism against a participant sharing a real experience was eliminated. The scenario comprising an older patient and their relative was described as a common scenario in the participants’ daily work.

*We were given the same … or similar conditions in that we all got to see one and the same information and the same film. I think it would have been much more difficult if we discussed and problematized a real patient case, because then, of course, we would approach the event a little differently. And we see different things … Yes, in this way, I think we probably became more unison.* (Interview No. 14)

The videos were described as highlighting different aspects of a complex situation that could then be discussed from different perspectives, which inspired the participants to jointly come up with workable solutions. After the ethics rounds, the participants described themselves as thinking of the videos and its following conversations, using them as an inner reference to compare with current and similar situations.

### Being surprised with new insights

The participants described how the ethics rounds surprised them by generating new insights. This was unexpected and a bit shocking, but still a positive experience. The ethics rounds triggered thoughts that raised awareness of shortcomings in their habitual behavior, which led to a willingness to change and to try to collaborate more in dialogue with others in the future. Furthermore, the ethics rounds contributed to an increased understanding that every problem may have a variety of different solutions. By participating in the ethics rounds, a feeling of being braver and less stressed in ethically demanding situations was experienced.

#### New awareness in the face of ethical problems

The participants described having a newfound awareness regarding ethical problems and their implications. The conversation in the ethics rounds made the ambulance clinicians see well-known problems from the perspective of others, which surprised them and made them consider their own ingrained behaviors with new eyes. They were also surprised by new insights regarding themselves, when realizing how their previous actions could have affected both patients, significant others, and colleagues in negative ways:

*I’m pretty fast, which can of course be very positive in certain situations, but sometimes I also run over people. And I know about this and of course I try to work on it, but sometimes you don’t think about it. But, in moments like this where you get to sit and discuss and listen to others, you can … Yes, you may be able to solve things in a different way than I usually do.* (Interview No 16)

Such insights were a shocking experience that gave the participants new ambitions for the continued work, where they wanted to be more cautious regarding their encounter with others and be more considerate regarding how they express themselves when talking to patients, significant others, and colleagues. At the same time, the ethics rounds signified a new awareness of other factors that affect their own behavior, such as how many hours they had worked and the time of day. This made them more forgiving of themselves, which, in turn, made them feel less stressed, based on the realization that a lack of energy can negatively affect a person’s abilities. The ethics rounds were further described as supporting new ways of thinking, which promoted more nuanced assessments:

*I can feel that after I participated in the ethics round, I brought this to my job in a way where I more consciously consider what kind of situation I end up in, and for whose sake do I do this and that …* (Interview No 4).

The ethics rounds provided the participants with new mental tools to use in ethically difficult situations. For instance, they described how they now took more time for reflection and involved the patient in the decision-making process, rather than making decisions for them. Having realized the need to involve the patient, the participants described themselves as being more confident and secure in their actions when they left patients at home, provided this was in accordance with the patient’s preferences. Furthermore, they described themselves as having understood the importance of asking for their colleague’s opinions, as the ethics rounds made them understand that their own perspective could be broadened by taking in others’ perspectives. With this awareness, the participants now described themselves as approaching care with greater thought and facing each new situation with an open mind instead of working automatically and without reflection. This approach made their work more enjoyable, as it involved more of a personal challenge to include other people’s perspectives before making decisions in ethically difficult situations. In addition, this approach also meant that they had gained the courage to stand up for their own opinions, even when other colleagues disagreed.

#### Shared learning through dialogue

The ethics rounds provided surprising insights into the importance of joint learning through dialogue with patients, significant others, and colleagues. Its structured dialogue showed a way to systematically map out important viewpoints and arrive at the solution to the problem that best matches the patient’s preferences. The participants described that the collegial dialogue on ethical problems had increased after the ethics rounds, where they together had learned and verbally practiced reasoning and analyzing ethical problems:

*We talk a lot more about the ethical problems and with that, I think you are a little more prepared. You start talking already in the car, then you get a bigger whole and a bigger picture of the patient before you even meet them and can discuss already there what can be ethical problems when you arrive, I think.* (Interview No 10)

This meant that the ambulance clinicians now possessed a language that enabled them to initiate and conduct ethical reasoning with their colleague already on the way out to the patient. Thus, they now felt more prepared when they encountered ethical problems. Also, colleagues who had not participated in the ethics rounds could benefit from them, as those who participated told them about their new experiences. In sharing these experiences, all employees could benefit from the ethics rounds, and thereby experience a new and common understanding of the value of solving difficult ethical problems through dialogue.

During the ethics rounds, it was considered an advantage if some participants had longer work experience, as this could make the dialogue more reflective. For employees with shorter experiences in the profession, this conveyed new knowledge by only listening. A subsequent joint reasoning could generate new knowledge and insights for all participants, regardless of their work experience, as this conveyed that there are often more solutions to an ethical problem than those that the individual can devise. Through the dialogue, participants were also surprised by listening to their colleagues:

*I had some wrongly preconceived notions about certain colleagues who I thought would think in a certain way. But in this forum, it turned out that they actually … Yes, they reasoned at a higher level than I thought they would.* (Interview No 16)

The experience of knowing more about how colleagues think was enriching and supportive when the regular written decision support manuals were not enough, which also helped in reducing the participants’ stress regarding their decision-making practices in ethically difficult situations.

## Discussion

The findings highlight that the success of ethic rounds depends largely on the facilitator’s competence and skills. The use of fictional pre-recorded video scenarios rather than a single participant’s experience-based case, helps the participants to reflect more deeply and freely. In being provided with these prerequisites, the participants expressed that they had been surprised with the new insights they had gained regarding themselves and others.

The findings indicate that the role of facilitator conveys a critical factor for the success of the ethics rounds process. The participants underline the importance of the facilitator’s ability to provide gentle guidance and the promotion of a permissive and open-minded atmosphere when discussing ethical problems. It was also found to be important that the facilitator firmly seeks responses from all participants and is permissive of the range of their responses and behaviors. Other qualitative studies (19, 29–30) have similarly found that it is important to facilitate an acknowledgement of different points of view, and an understanding that participants appreciate that everyone is encouraged to contribute and discuss in a non-polarizing manner. In congruence with our findings, [30] the importance of the facilitator’s neutrality is stressed, as this allows participants to respond to other parties involved in the ethical reflection, including their own team members. Further, the facilitator encouraged the participants to take the time to listen to others instead of trying to convince others of their own opinion. This allowed participants to structure the relevant arguments and adopt a more open attitude to other perspectives, rather than trying to discredit a colleague’s opinion [30]. However, there may be times when the facilitator fails to provide equal opportunity to everyone to articulate their views [29].

This points to the importance of ensuring that well-trained and responsive facilitators are enrolled in the ethics rounds process. In the present study, a trained facilitator was used. This seems to have contributed to a climate of a structured conversation that made it easier and more profound to reflect upon the various ethical dimensions in the video scenario that preceded the discussions. The present findings also show that the facilitator’s experience in leading discussions and having a solid knowledge base in ethics are important, as this allows the participants to trust the facilitator. Thus, the participants’ demands on the facilitator seem to align with what applies to ethics rounds, where the clinical ethicist is a well-educated facilitator with solid ethical skills [19, 23, 31]. The findings highlight the importance of the facilitators’ ability to clarify the content of the discussion and lead the discussion in a moderate, permissive, and kind way. This is in line with Silén’s [19] research, which also highlights the facilitator’s ability to cope with any conflicts that might arise. To reduce reliance on external experts and to train healthcare professionals in moral deliberation, another study [32] showed that practice and training are crucial aspects and contribute most to the development of necessary competencies. However, feeling competent is not one-dimensional. Although the facilitators may feel competent, they are also aware that they have a lot to learn to reduce uncertainty [32]. Among facilitators who have had different forms of training to cope with the task, there seems to be consensus that the facilitator has an important, but difficult, role, and one that needs to be supported [33]. In this role, participants emphasize that ethics facilitators should be respectful, responsive, accessible, and approachable [30].

As described by the study participants, the use of a filmed scenario as a starting point for discussion promotes a safe environment, generates a joint learning environment, and focuses on the solution to a problem that best matches the patient’s, significant others’, and colleagues’ preferences. The value of conducting collegial group deliberation is highlighted in a variety of health care settings, often taking its starting point as a complex working situation personally experienced by one of the participants [34]. The participants in the present study emphasize the advantages of discussing a fictitious case represented in a filmed scenario, stating the benefits that all participants were given the same pre-condition and a common starting point in the subsequent discussion. Another benefit to this approach is that, with a filmed scenario, the ethics round is guaranteed a valid case for discussion. This counteracts the common problem of participants who attend the ethics round without having had the time to prepare a suitable case to present to the group [19]. Also, the risk of criticism within the group or against a participant sharing a real experience was eliminated, which complies with others’ negative experiences of ethics rounds feeling as though they are attending a trial in court [35]. Using videos as a form of professional learning support within health care contexts has shown to enhance the understanding of the caring encounter by providing a deeper understanding of the complexity in a patient meeting [36]. Moreover, using a filmed scenario as a startup for reflection elaborates the participants’ opportunity to reflect on their own (pre-)understandings in relation to the understandings of others [36].

The findings of this study reveal that the ethic rounds surprised the participants with new insights into the complexity of ethical problems and their implications by challenging their preconceived notions. These findings correspond to the findings of Silén et al. [19], as well as the description of ethics rounds as a method for supporting healthcare personnel to develop ethical competencies and get insight into ethical issues [21]. Listening and discussing with others is an important aspect of learning in ethics rounds [20, 22].

The new insights were described by the participants as being shocking, particularly when realizing the impact of their actions on patients, colleagues, and others. At the same time, these insights created new ambitions and mental tools for their continued work as ambulance clinicians. In line with these experiences, earlier research describes a compassionate approach, one that includes listening and adhering to patients’ and other colleagues’ thoughts, to indicate the presence of an ethical competence [7].

The potential use of ethics rounds as a method and measure to prevent future ethical problems has earlier been raised by Svantesson et al. [9]. The participants in this study claim to have improved their ethical competence after participating in only one ethics round. However, Molewijk et al. [23] state that it is unknown whether these newly developed competencies obtained by participating in ethics rounds improved the quality of care and their effect in the clinical setting over time. There is disagreement in previous research about whether ethics rounds have a direct effect on clinical practice [19]. Nevertheless, the participants in our study described how their new insights prompted reflection and more collaborative decision-making as well as reducing stress in challenging ethical situations. However, we acknowledge that these findings should be reviewed carefully, considering that they are drawn on self-reported descriptions of the ethics rounds effect.

### Methodological considerations

The results of the study should be interpreted while considering some limitations. One limitation could be that the participants described their experiences of taking part in only one ethics round. However, the results were rich and nuanced. Another limitation might be that not all participants from the ethics rounds were interviewed, conveying a risk that only those with a positive experience of the rounds were included. A third limitation is that no emergency medical technicians participated in the interviews. Additionally, the time that passed between the ethics round and the data collection might be a limitation, allowing participants to forget their reflections. On the other hand, this might also have been beneficial, as participants therefore had the time to put their new insights into action. The interviews were conducted by four different interviewers, which may have influenced the results negatively, as different questions were therefore posed. Conversely, all four interviewers were experienced researchers, and familiar with collecting data in interviews. In addition, three pilot interviews were completed for training purposes before data collection began. The interviews were then analysed by two experienced qualitative researchers. In any qualitative study, researchers should be aware of, and attempt to bridle their pre-understandings. Therefore, discussions between all researchers were conducted throughout the process to prevent such pre-understandings from having an impact. Further, a constant dialogue took place between all researchers during the analysis, and the findings were discussed until consensus was reached. Trustworthiness was promoted by describing the research process carefully, to make it possible for others to follow and transfer the results to other settings. The findings of this study might be transferable to other ambulance contexts, or similar contexts where nurses often stand rather alone in the face of ethical problems, i.e., within primary care or in nursing homes. Moreover, the relation between patients and ambulance clinicians may vary across different countries and cultures. Therefore, similar studies in different contexts and cultures are warranted.

## Conclusion

In conclusion, structured ethics rounds seem to create opportunities for continuous development in ethical competence that may improve the quality of care in the ambulance service. Structured guidance and facilitated group reflections offer ambulance clinicians the opportunity to facilitate both personal and professional development, especially if the discussed scenario is pre-recorded and fictional, thus reducing the risk of any participant being personally criticized. An important prerequisite for the development of ethical competence is a well-educated facilitator who conducts a permissive and inclusive approach, with kindness and attentiveness. Consequently, this type of ethics rounds may be considered a useful pedagogical model for the development of ethical competence.

### Electronic supplementary material

Below is the link to the electronic supplementary material.

Supplementary Material 1

## Data Availability

No datasets were generated or analysed during the current study.
