# Supplementary material for: Ethics rounds in the ambulance service: a qualitative evaluation
Source: BMC Med Ethics. 2024 Jan 18;25:8. doi: 10.1186/s12910-024-01002-6 (PMC10795226; doi:10.1186/s12910-024-01002-6)
Supplement: Supplementary file 1 — Supplementary Material 1 [file 12910_2024_1002_MOESM1_ESM.docx]

# Interview Guide

1. **Approach**

Treat the interview as an open dialogue, utilizing the questions as a guide rather than a rigid questionnaire.

1. **Introduction**

Begin with demographic inquiries to establish contextual background.

1. **Main Focus**

Initiate the conversation with the question: How did you experience the ethics round?

1. **Exploration**

Elaborate on the participant's experience, allowing them to express their thoughts freely.

1. **Possible follow-up Questions**

Depending on the participant's responses, try to delve deeper into the impact of the ethics round concerning:

1. Ethical competence.
2. Ethical climate within the workplace.
3. Moral courage.
4. Stress.
5. Autonomy and paternalism.

Remember to maintain an open and non-directive approach, allowing participants the freedom to share their perspectives in-depth. The goal is to capture nuanced insights that contribute meaningfully to the exploration of the impact of ethics rounds on individuals and their professional environments.
